# A High-Calorie Diet Aggravates Lipopolysaccharide-Induced Pulmonary Inflammation in Juvenile Rats via Hypothalamic-Pituitary-Adrenal Axis-Related Pathways

**DOI:** 10.3390/ijms26146554

**Published:** 2025-07-08

**Authors:** Qianqian Li, Hui Liu, Chen Bai, Lin Jiang, Chen Su, Xueying Qin, Tiegang Liu, Xiaohong Gu

**Affiliations:** 1 School of Traditional Chinese Medicine, Beijing University of Chinese Medicine, Beijing 102488, China; liqianqian0725@163.com (Q.L.); bc@bucm.edu.cn (C.B.); 17888806672@163.com (L.J.); 15138907727@163.com (C.S.); 20220941180@bucm.edu.cn (X.Q.); 2 School of Traditional Chinese Medicine, Shandong University of Traditional Chinese Medicine, Jinan 250300, China; 3 Institute of Chinese Medicine Epidemic Disease, Beijing University of Chinese Medicine, Beijing 102488, China; 13671081286@163.com

**Keywords:** high-calorie diet, pneumonia, lung-brain axis, hypothalamic-pituitary-adrenal axis, hypoxia-inducible factor-1 alpha

## Abstract

The hypothalamic-pituitary-adrenal (HPA) axis plays an important regulatory role in inflammatory responses to systemic or local infection in the host. A high-calorie diet, which can aggravate pediatric pneumonia and delay recovery, is intimately associated with HPA axis disorder; however, its underlying mechanisms remain unknown. This study examined whether the mechanism by which a high-calorie diet aggravates pneumonia is related to HPA axis disorder. In this study, juvenile rats were fed a high-calorie diet and/or nebulized with lipopolysaccharide (LPS) for model construction. Our data shows that a high-calorie diet increases interleukin-1 beta(IL-1β), interleukin-6 (IL-6) and tumor necrosis factor-alpha (TNF-α) levels in lung tissues and aggravates LPS-induced inflammatory injury in the lungs of juvenile rats. Additionally, we found that a high-calorie diet decreases the expression level of serum adrenocorticotropic hormone (ACTH) and corticosterone (CORT) in juvenile rats with pneumonia, resulting in HPA axis disorder. Hypothalamus proteomics and Western blot results proved that a high-calorie diet upregulated the expression level of hypothalamus hypoxia-inducible factor-1 alpha (HIF-1α) in juvenile rats with pneumonia, and this mechanism is associated with reduced HIF-1α ubiquitination. We further observed that HPA axis disorder was significantly abated and inflammatory damage in rat lung tissues was significantly alleviated after in vivo HIF-1α pathway inhibition. This shows that pneumonia aggravation by a high-calorie diet is associated with interference in the HIF-1α-mediated HPA axis. A high-calorie diet boosts HIF-1α signaling in the hypothalamus and exacerbates LPS-induced pneumonia by disrupting the HPA axis. This sheds light on lung inflammation and strengthens the lung-brain connection.

## 1. Introduction

Pneumonia is a common acute respiratory tract infection, mainly caused by bacteria, viruses, and fungi [1]. It is a common pediatric disease and is also a leading cause of death in children globally [2,3,4]. The World Health Organization estimates that more than 150 million children aged <5 years develop pneumonia every year and nearly 2 million of them require hospitalization [5]. Although the development of vaccines and improvements in pediatric nutrition and feeding methods in recent years have resulted in a decrease in the incidence and mortality rate of pediatric pneumonia [6,7], it remains a global health challenge. Therefore, there is a need to continue in-depth studies on the pathogenesis of pediatric pneumonia and develop new treatment strategies.

Previous studies have found that the occurrence of respiratory tract infections is related to poor dietary habits [8]. A high-calorie Western diet will activate innate immunity and impair adaptive immunity, resulting in chronic inflammation and weakening of the host’s defense against respiratory pathogens [9]. Khanh Hoang N et al. found that a high-fat diet induced severe weight loss and lung inflammation in obese mice after lipopolysaccharide (LPS) administration [10]. Previous studies also proved that a high-calorie diet aggravates LPS-induced lung inflammatory damage [11,12]; however, the specific underlying mechanisms are yet to be elucidated.

The lung-brain axis is an emerging research field that is intimately associated with respiratory tract infections and nervous system diseases [13,14]. Moreover, the hypothalamic-pituitary-adrenal (HPA) axis is also one of the known or predicted potential lung-brain mechanisms [15]. The HPA axis consists of the paraventricular nucleus of the hypothalamus, pituitary frontal lobe, and adrenal glands and connects peripheral organs with the central nervous system [16]. The HPA axis releases glucocorticoid in response to stressful situations (such as infections) to supply the energy needed to maintain homeostasis. The effects of glucocorticoids on the immune system include inhibiting leukocyte and immune helper cell responses and pro-inflammatory cytokine synthesis [17]. Excessive or insufficient glucocorticoid release will result in severe harmful metabolic and immunological effects. Therefore, precise HPA axis activity regulation is vital [18].

The hypothalamus is the core of the HPA axis. According to previous reports, the hypothalamus is a critical nexus in the immune system–brain communication and disease signal transduction during inflammatory peripheral injury [19]. A previous study demonstrated that the hypothalamus plays an important role in the dissemination of corona virus disease 2019 infection in the host as the hypothalamus circuit is where the virus enters through the olfactory bulb and communicates with the important respiratory network. The virus also reaches the periphery through the HPA axis [20]. A high-calorie diet, such as a high-fat diet, will induce hypothalamus inflammation and change peripheral metabolism and behavior through inflammatory changes and glial cell recruitment [21]. Furthermore, a high-fat diet can also cause HPA axis dysregulation [22,23]. However, it is still unclear whether the mechanism by which a high-calorie diet aggravates pediatric pneumonia in children is related to HPA axis disorder. Therefore, this study aimed to examine the effects of a high-calorie diet on LPS-induced pulmonary inflammatory damage in juvenile rats with pneumonia and determine whether the specific mechanism is related to HPA axis disorder.

## 2. Results

### 2.1. A High-Calorie Diet Significantly Aggravates LPS-Induced Pneumonia in Juvenile Rats

**Figure 1:**
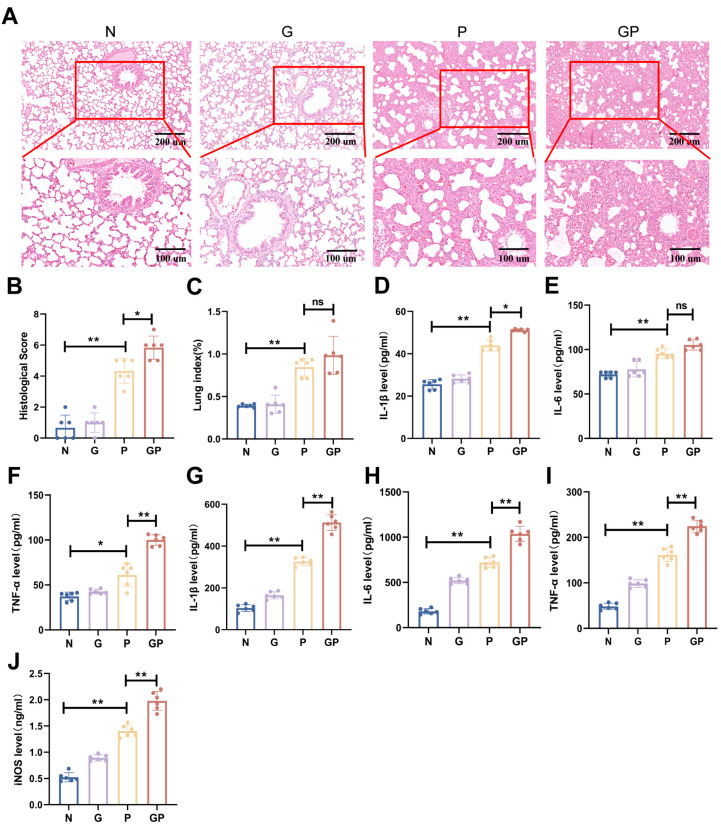
A high-calorie diet significantly aggravates lipopolysaccharide(LPS)-induced pneumonia in juvenile rats. (**A**) H&E staining (100×/200×). (**B**) Quantified scoring of pulmonary pathology. (**C**) The lung index of rats. (**D**–**F**) Serum IL-1β, IL-6, and TNF-α levels were quantified using ELISA. (**G**–**I**) IL-1β, IL-6, and TNF-α levels in lung tissues were quantified using ELISA. (**J**) Serum iNOS levels were quantified using ELISA. Data are represented as the mean ± SD (*n* = 6). * *p* < 0.05, ** *p* < 0.01, and ns (*p* > 0.05).

We evaluated the severity of lung inflammation injury via histopathologic analysis. In the N group, the alveolar wall structure was clear and complete, and there was no inflammatory infiltration of the alveoli. In the P group, the lung index was increased by thickened alveolar wall and inflammatory cell infiltration of alveoli and the interstitium (Figure 1A,B). Compared with the P group, rats in the GP group displayed severe lung damage indexed by massive inflammatory cell infiltration and alveolar structure destruction (Figure 1A). The lung pathological scores were higher (Figure 1B). We also measured the inflammatory cytokine levels and found that the levels of pro-inflammatory cytokines, such as interleukin-1 beta (IL-1β), interleukin-6 (IL-6), and tumor necrosis factor-alpha (TNF-α), were significantly increased after LPS challenge (Figure 1D–I). Serum IL-1β and TNF-α levels, along with lung tissue IL-1β, IL-6, and TNF-α levels, were further increased in the GP group compared with the P group (Figure 1D–I). Higher levels of inducible nitric oxide synthase (iNOS) were the symbol of acute pneumonia, which was caused due to diverse reasons [24]. Therefore, we measured serum iNOS levels, which were significantly elevated in the GP group compared to the P group (Figure 1J, Supplementary Table S1). Taken together, the results showed that a high-calorie diet could aggravate the LPS-induced lung inflammatory damage.

### 2.2. A High-Calorie Diet Disturbs the HPA Axis

**Figure 2:**
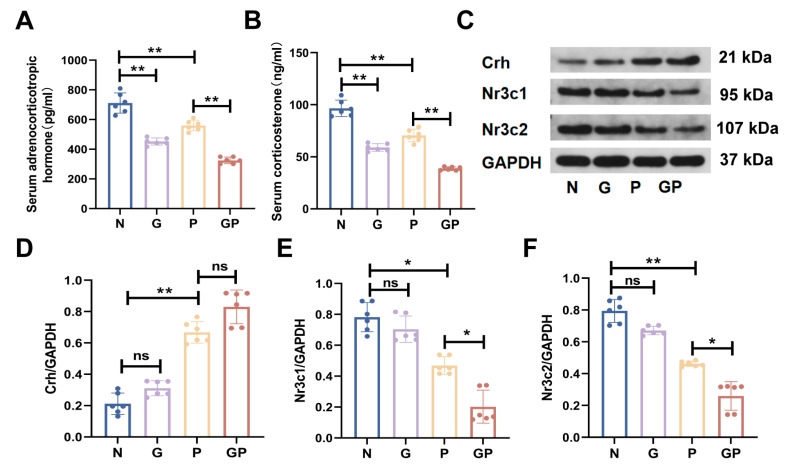
A high-calorie diet disturbs the hypothalamic-pituitary-adrenal (HPA) axis in rats with LPS-induced pneumonia. (**A**) The levels of adrenocorticotropic hormone in the serum using ELISA. (**B**) The levels of corticosterone in the serum using ELISA. (**C**) Representative Western blots of Crh, Nr3c1, and Nr3c2. (**D**) Quantification of the Crh protein level. (**E**) Quantification of the *Nr3c1* protein level. (**F**) Quantification of the Nr3c2 protein level. Data are presented as the mean ± SD (*n* = 6). * *p* < 0.05, ** *p* < 0.01, and ns (*p* > 0.05).

Our results showed that compared with the N group, the expression levels of adrenocorticotropic hormone (ACTH) and corticosterone (CORT) in peripheral blood significantly decreased in juvenile rats fed a high-calorie diet (Figure 2A,B, Supplementary Table S1), but this did not induce significant changes in the protein expression levels of corticotropin-releasing hormone (Crh), nuclear receptor subfamily 3 group c member 1 (*Nr3c1*), and nuclear receptor subfamily 3 group c member 2 (Nr3c2) in hypothalamus tissues (Figure 2C–F). Compared with the P group, there was no significant change in the expression level of Crh protein in the hypothalamus of the GP group rats (Figure 2C,D), while the protein expression levels of *Nr3c1* and Nr3c2 significantly decreased (Figure 2C,E,F) and those of ACTH and CORT in peripheral blood significantly decreased as well (Figure 2A,B). These data showed that the aggravation of LPS-induced pneumonia from a high-calorie diet may be due to its disruption of the HPA axis balance.

### 2.3. The Protein Expression in the Hypothalamic Tissue of GP Mice Is Different from That of Pure P Mice

**Figure 3:**
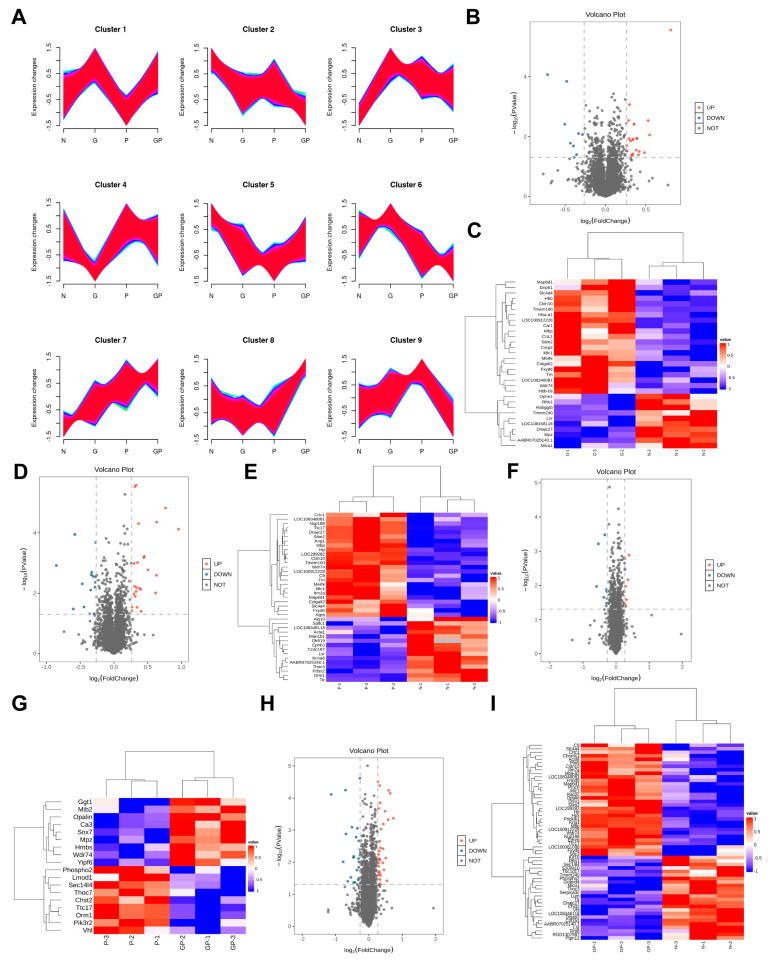
Differences in protein expression in the hypothalamic tissue between N, G, P, and GP rats. (**A**) The Mfuzz cluster analysis was performed on N, G, P, and GP groups proteomics. The color from blue to red indicates that the membership value of this protein increases, which represents the protein’s importance in the cluster to some extent. (**B**) The volcano map of the G group compared to the N group. (**C**) The differential proteins enrichment analysis clustering heatmap of the G group compared to the N group. (**D**) The volcano map of the P group compared to the N group. (**E**) Differential proteins enrichment analysis clustering heatmap of the P group compared to the N group. (**F**) The volcano map of the GP group compared to the P group. (**G**) The differential proteins enrichment analysis clustering heatmap of the GP group compared to the P group. (**H**) The volcano map of the GP group compared to the N group. (**I**) The differential proteins enrichment analysis clustering heatmap of the GP group compared to the N group.

To further analyze the mechanism by which a high-calorie diet exacerbates pneumonia, we used hypothalamus samples for a proteomics study. A total of 5629 proteins were identified through a quantitative proteomics analysis, of which 5623 were quantifiable proteins. Figure 3A shows the variation trends of all protein expression levels in the samples. We calculated the fold change (FC) of expression levels for various proteins between two groups of samples. Differentially expressed proteins (DEPs) were selected based on P < 0.05 and FC > 1.2-times (upregulated to more than 1.2-times or downregulated to less than 0.83). When the G group was compared with the N group, 33 DEPs were selected, of which 22 were upregulated proteins and 11 were downregulated proteins (Figure 3B,C). When the P group was compared with the N group, 40 DEPs were selected, of which 24 were upregulated proteins and 16 were downregulated proteins (Figure 3D,E). When the GP group was compared with the P group, 18 DEPs were selected, of which 9 were upregulated proteins and 9 were downregulated proteins (Figure 3F,G). When the GP group was compared with the N group, 57 DEPs were selected, of which 32 were upregulated proteins and 25 were downregulated proteins (Figure 3H,I).

### 2.4. A High-Calorie Diet Is Associated with Cellular Response to Corticosteroid Stimulus in the Hypothalamic Tissue of Juvenile Rats with LPS-Induced Pneumonia

**Figure 4:**
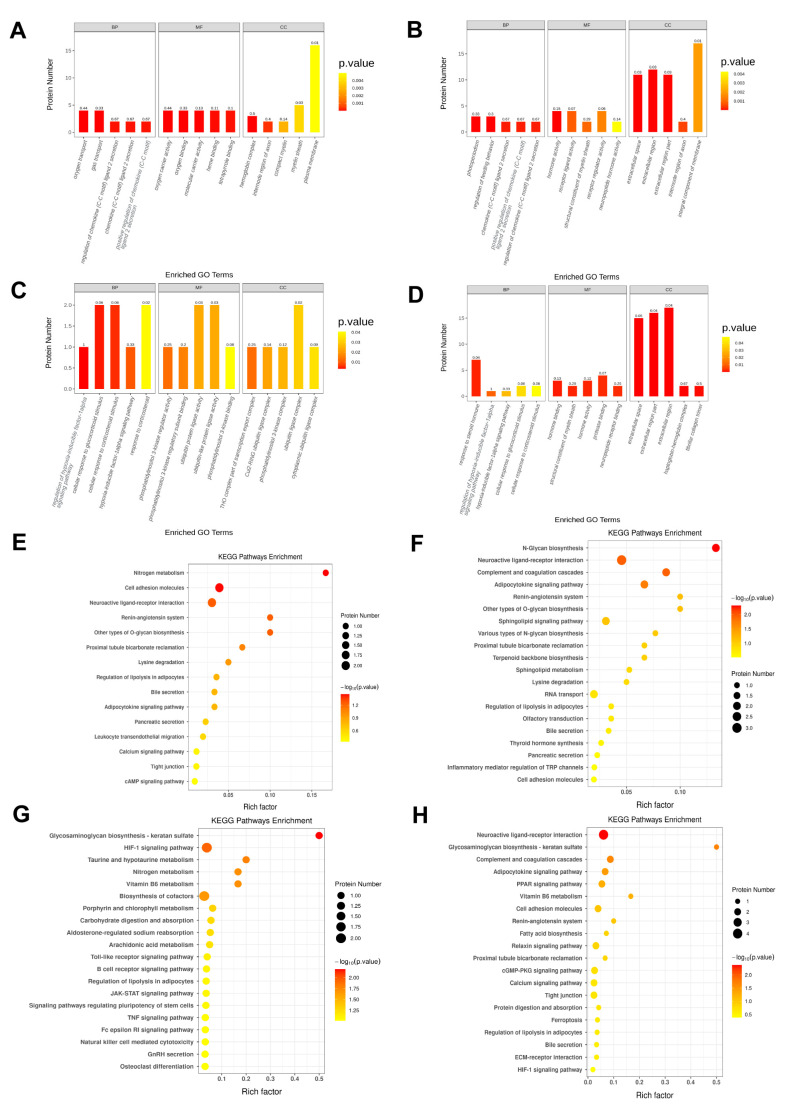
Gene ontology and kyoto encyclopedia of genes and genomes(KEGG) enrichment analyses. (**A**) Gene ontology of DEPs between the N and G groups. (**B**) Gene ontology of DEPs between the N and P groups. (**C**) Gene ontology of DEPs between the GP and P groups. (**D**) Gene ontology of DEPs between the N and GP groups. (**E**) KEGG enrichment analysis of DEPs between the N and G groups. (**F**) KEGG enrichment analysis of DEPs between the N and P groups. (**G**) KEGG enrichment analysis of DEPs between the GP and P groups. (**H**) KEGG enrichment analysis of DEPs between the N and GP groups.

The DEPs between groups GP and P represent the effects of a high-calorie diet on LPS-induced pneumonia to some extent. Therefore, we used the differentially expressed proteins between groups GP and P for downstream bioinformatics analyses to identify related mechanisms. First, we performed gene ontology enrichment analyses on the differentially expressed proteins between these two groups, and the results showed the following: At *p* < 0.05, there were 123 biological process (BP) entries, 15 cellular component (CC) entries, and 28 molecular function (MF) entries. BPs were mainly related to the regulation of the hypoxia-inducible factor-1 alpha signaling pathway, cellular response to glucocorticoid stimulus, cellular response to corticosteroid stimulus; CCs were mainly related to the Cul2-RING ubiquitin ligase complex, phosphatidylinositol 3-kinase complex, ubiquitin ligase complex, and cytoplasmic ubiquitin ligase complex; also, MFs were mainly related to carbon–oxygen lyase activity, leukotriene-C(4) hydrolase, peptidyltransferase activity, uroporphyrinogen-III synthase activity. As shown in Figure 4C, the top five entries were listed for each item.

We also carried out gene ontology enrichment analyses on DEPs in other groups. Gene ontology enrichment analyses of DEPs between the GP and N groups showed that biological processes were also related to the cellular response to glucocorticoid stimulus and cellular response to corticosteroid stimulus (Figure 4D). However, identical biological processes did not occur in the analysis of DEPs between the G and N groups and between the P and N groups. This suggests that the body’s responses to glucocorticoids and corticosteroids will change after LPS-induced pneumonia overlaps with a high-calorie diet. We also found previously that a high-calorie diet will cause serum ACTH and CORT to decrease (Figure 2E,F) and expression levels of hypothalamus *Nr3c1* and Nr3c2 proteins to decrease (Figure 2A,C,D) in juvenile rats with LPS-induced pneumonia. The above results suggest that a high-calorie diet may induce changes in the responses of the hypothalamus to glucocorticoids and corticosteroids in juvenile rats with LPS-induced pneumonia.

### 2.5. A High-Calorie Diet May Aggravate LPS-Induced Pneumonia by Activating the HIF-1α Pathway in the Hypothalamus

**Figure 5:**
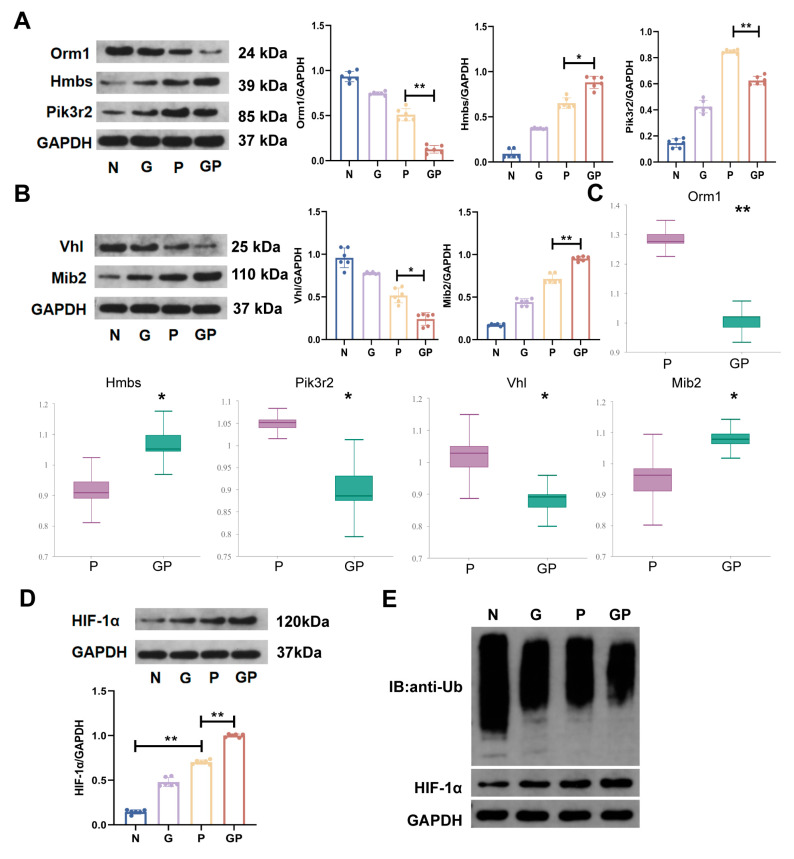
Validation of differentially expressed proteins (DEPs). (**A**) The levels of Orm1, Hmbs, and Pik3r2 in the hypothalamic tissues were evaluated by Western blotting. (**B**) The levels of VHL and Mib2 in the hypothalamic tissues were evaluated by Western blotting. (**C**) Differences in the expression of proteins between the GP and P groups in the proteome data. (**D**) The levels of HIF-1α in the hypothalamic tissues were evaluated by Western blotting. (**E**) The levels of HIF-1α ubiquitination in the hypothalamic tissues were evaluated by co-immunoprecipitation. Data are represented as the mean ± SD (*n* = 6). * *p* < 0.05, ** *p* < 0.01.

We carried out the kyoto encyclopedia of genes and genomes(KEGG) pathway enrichment analysis on DEPs in the GP/P groups, and the top 20 pathways are shown in Figure 4G. There were six pathways that satisfied *p* < 0.05, namely, glycosaminoglycan biosynthesis–keratan sulfate, HIF-1α signaling pathway, taurine and hypotaurine metabolism, vitamin B6 metabolism, nitrogen metabolism, and cofactor biosynthesis, which are mainly related to environmental information processing and metabolism. Biological processes in gene ontology enrichment analyses of DEPs in the GP/P groups suggest that they were associated with the regulation of the hypoxia-inducible factor-1α signaling pathway (Figure 4C). In addition, gene ontology and KEGG pathway enrichment analyses of DEPs in the GP/N groups also suggest that they were associated with the regulation of the HIF-1α signaling pathway (Figure 4D,H). Interestingly, the DEPs in the G/N and P/N were not associated with this pathway (Figure 4E,F). The above results show that the addition of a high-calorie diet in juvenile rats with pneumonia will affect the HIF-1α signaling pathway in the hypothalamus. This somewhat suggests that the aggravation of LPS-induced pneumonia by a high-calorie diet is related to interference with the hypothalamus HIF-1α signaling pathway. To prove the reliability of the proteomics results, we used the Western blot method to validate five important DEPs in the GP/P group (Figure 5A,B), and the Western blot results were the same as the proteomics ones.

Following that, we validated the HIF-1α signaling pathway by testing the protein expression level of HIF-1α in hypothalamus tissues. Compared with the P group, a high-calorie diet increased the protein expression level of HIF-1α in the hypothalamus in the GP group (Figure 5D). Gene ontology enrichment analyses of DEPs between the GP and P groups showed that cellular components were mainly associated with the Cul2-RING ubiquitin ligase complex, ubiquitin ligase complex, and cytoplasmic ubiquitin ligase complex (Figure 4C). Protein validation results showed that a high-calorie diet caused the protein expression level of Von Hippel-Lindau (VHL) in the hypothalamus in the GP group to decrease (Figure 5B). A decrease in the VHL level can decrease HIF-1α ubiquitination and degradation, thereby causing HIF-1α accumulation. We further measured the ubiquitination level of HIF-1α, and the results showed that a high-calorie diet caused the ubiquitination level of HIF-1α in the hypothalamus in the GP group to decrease (Figure 5E). These results suggest that a high-calorie diet may promote HIF-1α expression in hypothalamus tissues, which may be associated with impaired HIF-1α ubiquitination and degradation.

### 2.6. The Aggravation of LPS-Induced Pneumonia by a High-Calorie Diet Is Associated with the HIF-1α-Mediated HPA Axis

**Figure 6:**
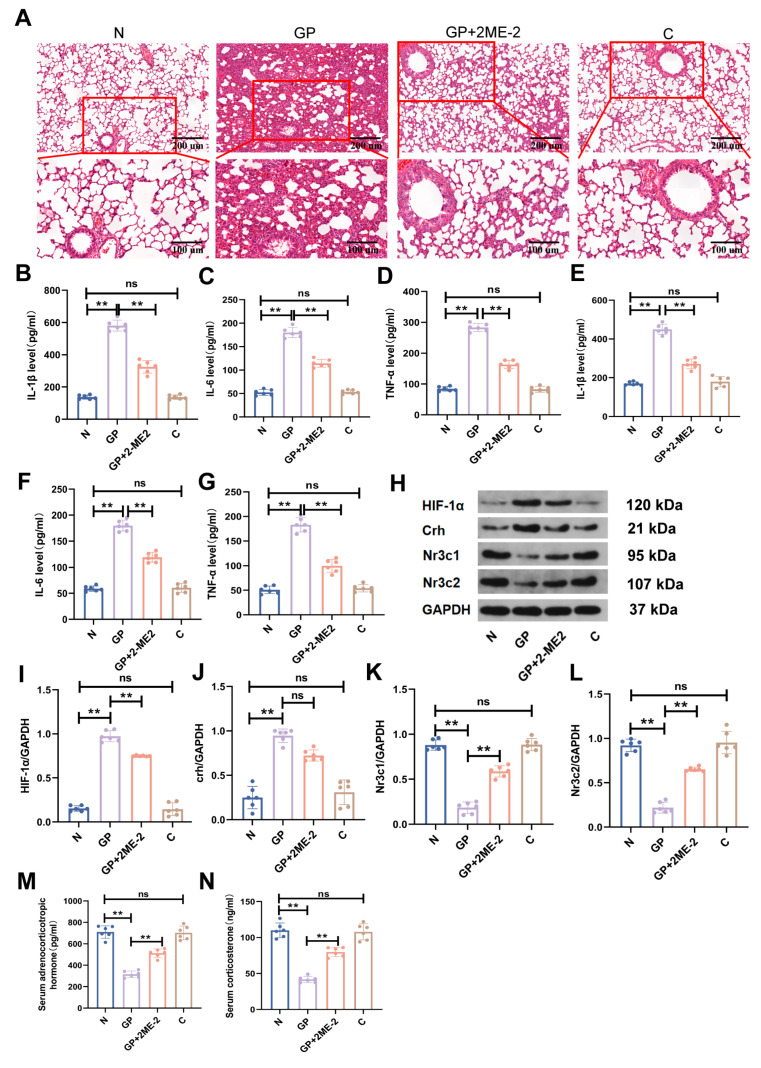
The aggravation of LPS-induced pneumonia by a high-calorie diet is associated with the HIF-1α-mediated HPA axis. (**A**) H&E staining (100×/200×). (**B**–**D**) The levels of IL-1β, IL-6, and TNF-α in lung tissues were evaluated using ELISA. (**E**–**G**) The serum levels of IL-1β, IL-6, and TNF-α were evaluated using ELISA. (**H**–**L**) The levels of HIF-1α, Crh, *Nr3c1*, and Nr3c2 in the hypothalamic tissues were evaluated by Western blotting. (**M**,**N**) The levels of ACTH and CORT in the serum were measured using ELISA. Data are represented as the mean ± SD (*n* = 6). ** *p* < 0.01, and ns (*p* > 0.05).

To further validate whether HPA axis changes caused by a high-calorie diet in juvenile rats with LPS-induced pneumonia were associated with the hypothalamus HIF-1α signaling pathway, we used an HIF-1α inhibitor to treat rats in the GP + 2ME-2 group. The results showed that compared with the GP group, lung inflammatory damage in the GP + 2ME-2 group was significantly alleviated (Figure 6A). Compared with the GP group, the expression levels of pro-inflammatory factors such as IL-1β, IL-6, and TNF-α in the serum and lung tissues in the GP + 2ME-2 groups were significantly decreased (Figure 6B–G, Supplementary Table S2). At the same time, compared with the GP group, there was no significant difference in the Crh expression level in the hypothalamus, and *Nr3c1* and Nr3c2 expression levels significantly increased in the GP + 2ME-2 groups (Figure 6H,J–L, Supplementary Table S2). Serum ACTH and CORT levels in the GP + 2ME-2 groups were also significantly upregulated (Figure 6M,N, Supplementary Table S2). We found that HPA axis disorder was alleviated when HIF-1α expression in the hypothalamus was inhibited and lung inflammatory injury in rats improved. This indicates that the inhibition of HIF-1α expression in the hypothalamus can somewhat reverse the aggravation of pneumonia via a high-calorie diet. This suggests that the aggravation of pneumonia caused by a high-calorie diet may be associated with hypothalamus HIF-1α-mediated HPA axis disorder.

## 3. Discussion

Increased income and urbanization have led to changes in global dietary structure, with traditional diets being replaced by high-calorie diets and highly processed foods such as high-glucose and high-fat foods [25,26]. Consumption of a high-calorie diet is common in children due to factors such as children themselves, parents, and schools, particularly high-energy and low-nutrition beverages, which contribute a large amount of calories and are low in nutrients [27]. A study showed that the mean energy intake of 2–18-year-old adolescents is 1870 kilocalories [28], and high-energy and low-nutrient foods have led to an increase in the prevalence of noncommunicable diseases [29]. Diets affect the health of the population and contribute to the development of respiratory diseases, and nutrition therapy is crucial to improving the respiratory micro-ecosystem and pulmonary health function [26]. For example, a high-calorie diet can promote asthma progression, and decreasing caloric intake in the diet may help alleviate asthma symptoms [30]. More importantly, our clinical study found that a high-calorie diet is intimately associated with recurrent respiratory tract infections in children [8]. Currently, there is a paucity of studies on the mechanisms by which a high-calorie diet affects pediatric pneumonia. Therefore, this study designed an experimental model to mimic unhealthy dietary patterns in children (characterized by high calorie, high carbohydrate, high fat, high sodium, and low fiber intake) to evaluate the mechanistic effects of such a diet on childhood pneumonia. In the existing animal experiments, high-fat or high-calorie diet interventions mostly last two weeks or several months. However, a short-term high-calorie diet is more common in children, and the effects of such a diet on the body are often ignored. Therefore, we focused on the effects of a short-term high-calorie diet on the body’s response to pneumonia. As expected, a short-term high-calorie diet increased IL-1β, IL-6, and TNF-α levels in lung tissues and elevated serum levels of IL-1β, TNF-α, and iNOS expression, thereby aggravating pulmonary inflammatory injury in juvenile rats.

The lung-brain axis involves the nervous system, endocrine system, immune system, microorganisms and metabolites, and gases [31]. The close relationship and functional integration between the respiratory and endocrine systems may be essential for lung development and the maintenance of homeostasis and defense responses [32]. The HPA axis is an important component of the endocrine pathway and an important pathway for achieving lung-brain communication [32]. The HPA axis plays an important role in stress responses and host defense against infection. HPA axis inhibition causes cortisol responses to decrease, which may lead to stress response injury and insufficient host defense against infection [33]. Currently, there is controversy over the effects of a high-fat diet on the HPA axis. A systematic review demonstrated that intake of a high-fat diet for at least two consecutive weeks has limited effects on baseline HPA axis activity in mice [34]. Another study demonstrated that 12 weeks of a high-fat diet significantly increased baseline plasma cortical levels but did not increase the immune responsiveness of glucocorticoid receptors in the hypothalamus [35]. Another study showed that a high-fat diet could decrease HPA axis activity, and both short-term (1 week) and long-term (12 weeks) high-fat diet consumption could decrease plasma CORT level, while the transcriptional level of Crh in the paraventricular nucleus of the hypothalamus was increased with long-term high-fat diet consumption [36]. Our data show that a short-term high-calorie diet would decrease the serum CORT and ACTH levels but would not significantly change hypothalamus Crh, *Nr3c1*, and Nr3c2 protein expression levels, which was consistent with the findings reported by Auvinen HE et al. [36]. We also found that compared with rats in the P group, there was no significant change in the protein expression level of Crh in the hypothalamus of the rats in the GP group, while the protein expression levels of *Nr3c1* and Nr3c2 decreased. Corticosteroids play a negative feedback role when they act on multiple mineralocorticoid receptors (MRs) and/or glucocorticoid receptors (GRs) in the cerebrum and pituitary gland [37]. *Nr3c1* is a GR gene that plays a role in the HPA axis [38]. In the classical negative feedback loop, glucocorticoids bind to GRs (encoded by the *Nr3c1* gene) to inhibit the release of Crh and ACTH, thereby limiting the increase in glucocorticoids. Reduced *Nr3c1* expression seems to be associated with HPA axis activation by decreasing glucocorticoid inhibition and increasing Crh secretion [39]. A previous study demonstrated that low glucocorticoid exposure may cause more extensive inhibition of pathways downstream of ACTH secretion, and the efficacy of Crh in stimulating ACTH secretion is far greater than that of other stimuli [37]. This may adequately explain the changes in the ACTH content in peripheral blood in our study. Therefore, our study shows that the aggravation of LPS-induced pulmonary inflammatory damage in juvenile rats with pneumonia by a high-calorie diet may be related to HPA axis disorder.

We identified 18 DEPs between the GP and P groups through proteomics, visualized via volcano plots and heatmaps. Notably, key HPA axis hormones such as Crh, ACTH, and CORT were absent among these DEPs and were also undetected within the full set of 5629 identified proteins. This absence may be attributed to low protein abundance or small molecular weight. The DEPs primarily included upregulated proteins (Opalin, Ca3, Hmbs, Ggt1, Wdr74, Yipf6, Mpz, Mib2, Snx7) and downregulated proteins (Thoc7, Pik3r2, Lmod1, Chst2, VHL, Ttc17, Phospho2, Orm1). Gene ontology enrichment analyses of differentially expressed proteins between the GP and P groups showed that biological processes were related to the cellular response to glucocorticoid stimulus and cellular response to corticosteroid stimulus. Corticosteroids are key hormones of the HPA axis, among which glucocorticoids are closely associated with HPA axis regulation [40]. This suggests that a high-calorie diet may induce changes in the responses of the hypothalamus to glucocorticoids and corticosteroids in juvenile rats with LPS-induced pneumonia.

TMT proteomics analyses indicated that the HIF-1α signaling pathway was specific when the GP group was compared with the P group. Our further validation found that LPS nebulization alone can increase the protein expression level of HIF-1α in the hypothalamus, while a high-calorie diet further increases that level in the hypothalamus of rats with pneumonia. According to another previous study, a short-term seven-day high-fat diet could increase the protein expression level of HIF-1α in the hypothalamus, while a long-term 28-day high-fat diet significantly increased the protein expression level of HIF-1α, and its mechanism may be associated with the abnormal regulation of HIF-1α ubiquitination [41]. This is similar to the findings of our study. Per the existing literature, under normoxic conditions, proline hydroxylated HIF-1α binds to VHL E3 ubiquitin ligase, thereby triggering the ubiquitin/proteasome pathway to cause its rapid degradation [42]. Our study found that compared with the P group, the VHL protein expression and HIF-1α ubiquitination level in the GP group were decreased, suggesting that an increase in the HIF-1α protein expression level may be due to a decrease in the HIF-1α ubiquitination level caused by a decrease in the VHL protein expression level. Therefore, studies have shown that a high-calorie diet can upregulate the HIF-1α expression level in the hypothalamus of rats with LPS-induced pneumonia, and its mechanism may be associated with decreased HIF-1α ubiquitination.

HIF plays an important role in hypothalamic regulation and has nutrient sensing, hormone induction, metabolism regulation, and appetite regulation functions [43]. There is some crosstalk between glucocorticoids and the HIF signaling pathway. In zebrafish larvae, upregulated HIF-1 signaling inhibits GR and cortisol levels [44]. In adult rats, an increased HIF-1α expression level in the pituitary will promote the expression of the *CRHR1* gene [45]. Currently, the correlation between the hypothalamus HIF-1 signaling pathway and the HPA axis is still unknown. We observed that a high-calorie diet increased the HIF-1α expression level in the hypothalamus in rats with LPS-induced pneumonia and resulted in HPA axis imbalance. To further investigate whether HPA axis disorder induced by a high-calorie diet is associated with an upregulated HIF-1α expression level, we used an HIF-1α inhibitor to examine the correlation between the two. The results showed that compared with the GP group, plasma CORT and ACTH levels were significantly increased, and GR expression levels were also increased in the HIF-1α inhibitor group, but there was no significant change in the Crh expression level. These results suggest that HPA axis disorder caused by a high-calorie diet in juvenile rats with LPS-induced pneumonia may be associated with the activation of the HIF-1α signaling pathway.

## 4. Materials and Methods

### 4.1. Chemicals and Reagents

LPS was obtained from Sigma (St. Louis, MO, USA). Rat IL-1β/IL-6/TNF-α ELISA kits, Mouse NOS2/iNOS ELISA kits, QuicKey Pro Rat CORT ELISA kits, and Rat ACTH ELISA kits were supplied by Elabscience Biotechnology Co., Ltd. (Wuhan, China). cDNA Reverse Transcription Kits were obtained from Thermo Fisher Scientific (Waltham, MA, USA). TRIzol reagent was supplied by Invitrogen (Austin, TX, USA). SYBR Green PCR Master Mix was purchased from Bio-Rad Laboratory (Richmond, CA, USA). The BCA protein assay kit was purchased from Beyotime Biotechnology Co., Ltd. (Shanghai, China). Antibodies against HIF1-α and ubi were obtained from Proteintech Group (Chicago, IL, USA). Antibodies against Orm1, Mib2, Hmbs, Vhl, Crh, *Nr3c1*, and Nr3c2 were obtained from Affinity Biosciences (Columbus, OH, USA). Antibodies against Pik3r2 were obtained from Abcam (Cambridge, MA, USA). Antibodies against GAPDH were supplied by Hangzhou Xianzhi Biological Co., Ltd. (Hangzhou, China). Antibodies against HRP-conjugated anti-rabbit or anti-mouse secondary antibodies were supplied by Boster Biological Technology Co., Ltd. (Wuhan, China). HIF-1α (D1S7W) XP^®^ Rabbit mAb was obtained from Cell Signaling Technology (Danvers, MD, USA). Protein A+G Agarose was supplied by Beyotime Biotechnology Co., Ltd. (Shanghai, China). Phusion^®^ High-Fidelity PCR Master Mix was obtained from New England Biolabs (Essex, NJ, USA). AxyPrep DNA Gel Extraction Kit was purchased from Axygen Biosciences (Union City, CA, USA).

### 4.2. Animal Experiments

Three-week-old male SPF grade SD rats weighing 80 ± 10 g were purchased from Beijing Vital River Laboratory Animal Technology Co. Ltd., with an approval certificate number of SCXK (Beijing) 2021-0006 (Beijing, China) and an animal qualification certificate number of 110011231108478727. All rats were housed in the animal facility of the Beijing University of Chinese Medicine and given access to food and water ad libitum. Twenty-four three-week-old SD rats were acclimatized for three days before randomization into the following four groups: normal control group (N), pneumonia group (P), high-calorie diet group (G), and high-calorie diet + pneumonia group (GP). The maintenance feed and high-calorie feed for rats in this study were produced and underwent quality control by SPF (Beijing) Biotechnology Co., Ltd. Details of feed components are shown in Table 1 [12]. As shown in Table 2, rats in the G and GP groups were fed a high-calorie diet during the experiment, while those in the N and P groups were given the maintenance feed. From Day 4 of the experiment, LPS solution (0.5 mg/mL) was used for 30 min of nebulization twice daily in the P and GP groups. An equal volume of pure water was used for nebulization in the N and G groups. The general conditions of the rats were observed, and rats were weighed each day. On Day 7 of the experiment, 2% sodium pentobarbital (0.1 mL/10 g) was administered by intraperitoneal injection for anesthesia. Blood, hypothalami, and lung tissues were collected for further analysis. The lung tissues of the rats were rinsed with physiological saline to remove residual blood. After blotting excess surface moisture with absorbent paper, the wet weight of the tissues was recorded in grams (g). The lung index (%) was calculated as follows: [lung wet weight (g)/body weight (g)] × 100. The animal experiment procedures and animal care techniques were approved by the Institutional Animal Care and Use Committee of the Beijing University of Chinese Medicine (animal experiment ethics review No.: BUCM-2023082501-3056).

### 4.3. Lung Histopathology

After fixation with 4% paraformaldehyde for 24 h, the lung tissues were dehydrated and embedded in paraffin and then sliced into 5 μm thick sections. Lung tissue sections were stained with hematoxylin and eosin (H&E). Afterward, they were observed and photographed using an optical microscope (Nikon, Tokyo, Japan). A pathologist blinded to the treatment conducted the histological assessments. The score was graded according to the sum of the score for degree of damage such as hemorrhage, the number of infiltration cells, and edema. Each histological characteristic was assigned a score ranging from 0 to 3 [46].

### 4.4. TMT Proteomic Analysis

The proteomic analysis was performed on hypothalamus tissues from rats in the N, G, P, and GP groups. SDT (4% SDS, 100 mM Tris-HCl, 1 mM DTT, pH 7.6) buffer was used for sample lysis and protein extraction. The bicinchoninic acid (BCA) protein assay kit (Bio-Rad, USA) was used to quantitate protein content. For each sample, 20 µg of protein was mixed with 5× loading buffer and boiled for five minutes. The proteins were resolved on a 12.5% SDS-PAGE gel (constant current: 14 mA, 90 min). Coomassie blue R-250 staining was performed to visualize protein bands. The TMT reagent was used to label the 100 μg peptide mixture in each sample. Samples underwent vacuum centrifugation followed by the LC analysis. The MASCOT engine (Matrix Science, London, UK; version 2.2) in the Proteome Discovery 1.4 software program was used to search for the MS raw data of each sample for identification and quantitative analysis. The data were analyzed on a free online platform (https://bio-cloud.aptbiotech.com, accessed on 27 November 2023).

### 4.5. Drug Administration

The treatment of rats in the N and GP groups was the same as described above. We dissolved an HIF-1α inhibitor (2ME-2) in 1% dimethyl sulfoxide (DMSO), 40%PEG300, 5% Tween-80, and 54% saline. 2ME-2 (5 mg/kg; MedChemExpress, Monmouth Junction, NJ, USA, HY-12033) was injected intraperitoneally into the GP + 2ME-2 group once daily [47,48]. The animals in the vehicle control group (C) were injected intraperitoneally with equal amounts of the vehicle (1% DMSO, 40%PEG300, 5% Tween-80, and 54% saline) at the same time point. The animals in the N and GP groups were injected once daily with the same dose of physiological saline. All other experimental procedures applied to group C were identical to those implemented in group N.

### 4.6. Western Blot Analysis

The Western blot analysis was performed on hypothalamus tissues from rats. We applied a homogenizer with radio-immunoprecipitation assay solution to separate total proteins in the lung tissues, after which we measured the concentrations using a BCA protein assay kit. Proteins were separated on 12% SDS-PAGE gels at 80–120 V for 1.5 h and then transferred onto polyvinylidene difluoride (PVDF) membranes. The membranes were blocked with 5% skim milk at room temperature for 2 h. Then, the membranes were incubated with primary antibodies (1:1000) at 4 °C overnight. Thereafter, the membranes were incubated with HRP-conjugated secondary antibodies (1:5000) for 1 h at room temperature and detected using the enhanced chemiluminescence Western blotting detection reagent. The target protein bands were analyzed using the BandScan software program, version 5.0.

### 4.7. ELISA

The levels of IL-1β, IL-6, and TNF-α in serum and lung tissue, along with CORT, ACTH, and iNOS in serum, were assayed using ELISA kits following the manufacturer’s instructions.

### 4.8. Co-Immunoprecipitation (Co-IP)

The hypothalamus tissues were lysed in IP lysis buffer on ice for 10 min. Lysates were centrifuged at 10,000 rpm for 10 min at 4 °C, and the supernatant was collected as total protein. After determining the protein concentrations using a BCA protein assay kit, the protein concentration was adjusted to 1 mg/mL. To remove the non-specific binding, prewashing with 60 μL of protein A+G agarose beads was performed. After incubation with rotation at 4 °C for 2 h, the prepared lysates were centrifuged at 3000 rpm for five minutes at 4 °C and the supernatant was collected. The antibodies of HIF1a were added to the precleared lysates by overnight incubation at 4 °C, and then a 60 μL aliquot of protein A+G agarose beads was added to the mixture by incubation with rotation at 4 °C for 6 h. After centrifugation at 3000 rpm for five minutes, the supernatant was discarded, and the sediment was washed three times with precooled PBS before Western blotting.

### 4.9. Statistical Analysis

All experimental data analyses were completed using GraphPad Prism 9.3.0 Software (La Jolla, CA, USA). Quantitative data are presented as the mean ± standard deviation (SD) and were representative of at least three independent experiments. For data meeting the assumptions of normality and homogeneity of variance, multi-group comparisons were performed using one-way ANOVA; otherwise, non-parametric tests were applied. *p* < 0.05 was considered statistically significant.

## 5. Conclusions

**Figure 7:**
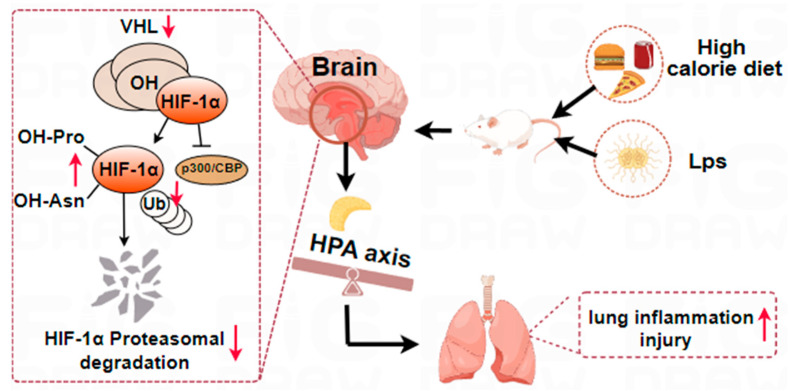
Schematic diagram of the mechanism by which a high-calorie diet exacerbates pneumonia. ↑, upregulation; ↓, downregulation.

We demonstrated that a high-calorie diet aggravated the lung inflammatory response in a rat model of LPS-induced pneumonia. We provided evidence that the mechanism by which a high-calorie diet aggravates pneumonia is associated with the HPA axis-related pathways (Figure 7). Our study results indirectly show the importance of a healthy diet for pneumonia patients. In addition, it partly provides new evidence for the lung-brain axis.

## 6. Limitations

Given that the effects of diet on the HPA axis are influenced by rodent strains, dietary composition, feeding duration, and other experimental variables [34], which results in inconsistent findings across the existing literature, our results can only partially reflect the impact of high-calorie diets on the HPA axis. Future investigations will examine the effects of high-calorie diets administered at different time points (e.g., 3, 7, 14, and 28 days) on the HPA axis and pneumonia in juvenile rats. Despite these limitations, our findings provide novel insights into the pathogenesis of pulmonary inflammatory injury and further evidence supporting the lung-brain axis.

## Figures and Tables

**Table 1:** The ingredients and nutrition of fodder.

	Rat Maintenance Fodder	High-Calorie Fodder
Energy, kJ/100 g	340	1828.12
Moisture	9.7%	10.10%
Crude protein	16.26%	13.73%
Crude fat	4.62%	16.10%
Carbohydrate	5.25%	58.80%
Sodium	0.22%	0.44%
Fiber	2.3%	1.8%
Crude ash	6.2%	0.83%
Calcium	1–1.8%	0.95%
Total phosphorus	0.6–1.2%	0.38%

**Table 2:** Experimental design.

Group	N	Feed (1–6 Days)	Atomization (4–6 Days)
N	6	Rat maintenance fodder	Physiologic saline
P	6	Rat maintenance fodder	LPS solution
G	6	High-calorie fodder	Physiologic saline
GP	6	High-calorie fodder	LPS solution

## Data Availability

The authors state that the all associated data are available in the manuscript.

## Supplementary Materials

The following supporting information can be downloaded at: https://www.mdpi.com/article/10.3390/ijms26146554/s1.

## Abbreviations

HPA	Hypothalamic-pituitary-adrenal
LPS	Lipopolysaccharide
ACTH	Adrenocorticotropic hormone
CORT	Corticosterone
HIF-1α	Hypoxia-inducible factor-1alpha
IL-1β	Interleukin-1 beta
IL-6	Interleukin-6
TNF-α	Tumor necrosis factor-alpha
Crh	Corticotropin-releasing hormone
*Nr3c1*	Nuclear receptor subfamily 3 group C member 1
Nr3c2	Nuclear receptor subfamily 3 group C member 2
VHL	Von Hippel-Lindau
MR	Mineralocorticoid receptors
GR	Glucocorticoid receptors
SPF	Specific Pathogen Free
iNOS	Inducible nitric oxide synthase
KEGG	Kyoto encyclopedia of genes and genomes
FC	Fold change
DEPs	Differentially expressed proteins
