# Supplementary material for: A High-Calorie Diet Aggravates Lipopolysaccharide-Induced Pulmonary Inflammation in Juvenile Rats via Hypothalamic-Pituitary-Adrenal Axis-Related Pathways
Source: Int J Mol Sci. 2025 Jul 8;26(14):6554. doi: 10.3390/ijms26146554 (PMC12296011; doi:10.3390/ijms26146554)
Supplement: Supplementary file 1 [file ijms-26-06554-s001.zip › ijms-3645393-supplementary.pdf]

A High-Calorie Diet Aggravates Lipopolysaccharide-Induced Pneumonia by Disturbing Hypothalamic-Pituitary-Adrenal Axis via the Hypoxia-Inducible Factor-1 Alpha Signaling Pathway

Qianqian Li [1,3,#], Hui Liu [2,#] ,Chen Bai [1], Lin Jiang [1], Chen Su [1], Xueying Qin [1], Tiegang Liu [1,*] and Xiaohong Gu [1,2,*]

Supplementary Table S1 Measured Parameters in Groups N, G, P, and GP

	N	G	P	GP
Histological Score	0.6667 ± 0.8165	1 ± 0.6325	4.333 ± 0.8165	5.833 ± 0.7528
Lung index(%)	0.3911 ± 0.0189	0.4079 ± 0.1081	0.8458 ± 0.1046	1.002 ± 0.1522
Serum IL-1β	25.59 ± 2.224	28.15 ± 1.890	43.92 ± 2.601	51.14 ± 0.6198
Serum IL-6	72.08 ± 3.537	77.74 ± 8.678	95.35 ± 5.317	105.2 ± 5.765
Serum TNF-α	37.28 ± 4.727	42.68 ± 2.623	61.15 ± 12.68	100.0 ± 5.978
lung IL-1β	103.8 ± 16.46	164.3 ± 16.91	325.5 ± 19.09	512.3 ± 37.77
lung IL-6	181.0 ± 29.85	521.2 ± 35.96	720.8 ± 54.66	1037 ± 84.93
lung TNF-α	48.17 ± 7.055	98.50 ± 8.432	161.7 ± 15.16	224.5 ± 13.07
Serum iNOS	0.5237 ± 0.08726	0.8917 ± 0.05922	1.404 ± 0.1136	1.978 ± 0.1794
ACTH	711.4 ± 68.50	452.2 ± 24.32	559.3 ± 35.73	325.0 ± 22.04
CORT	96.59 ± 7.858	58.93 ± 3.592	70.50 ± 6.081	38.73 ± 1.637
Crh	0.2117 ± 0.06876	0.3108 ± 0.04819	0.6670 ± 0.06835	0.8305 ± 0.1077
Nr3c1	0.7822 ± 0.09444	0.7042 ± 0.08538	0.4690 ± 0.05656	0.2023 ± 0.1071
Nr3c2	0.7943± 0.0723	0.6713± 0.02682	0.4623 ± 0.01661	0.2597 ± 0.0896
Orm1	0.9327 ± 0.05682	0.7450 ± 0.01914	0.5093 ± 0.06766	0.1250 ± 0.04245
Hmbs	0.09233 ±0.04832	0.3707±0.006282	0.6523±0.05793	0.8817±0.06810
Pik3r2	0.1453 ± 0.03379	0.4247 ± 0.04784	0.8477 ± 0.01385	0.6250 ± 0.03316
Vhl	0.9587 ± 0.1157	0.7777 ± 0.01218	0.5173 ± 0.08704	0.2407 ± 0.07376
Mib2	0.1737 ± 0.01313	0.4417 ± 0.04121	0.7143 ± 0.05687	0.9530 ± 0.02797
HIF-1α	0.1443 ± 0.02326	0.4793 ± 0.04992	0.7030 ± 0.02427	1.000 ± 0.01735

Supplementary Table S2 Measured Parameters in Groups N, GP, GP+2ME-2, and C

	N	GP	GP+2ME-2	C
lung IL-1β	137.5 ± 13.08	581.1 ± 34.19	324.9 ± 38.54	137.0 ± 13.58
lung IL-6	52.39 ± 6.254	179.9 ± 10.64	114.5 ± 8.390	53.47 ± 4.484
lung TNF-α	50.91 ±7.753	182.9 ±14.41	99.84 ±13.20	54.14 ±7.458
Serum IL-1β	170.2 ± 9.164	449.9 ± 25.72	271.6 ± 25.11	179.6 ± 26.87
Serum IL-6	59.14 ± 5.139	180.0 ±10.55	119.3 ±9.221	60.65 ± 8.766
Serum TNF-α	84.41 ± 7.361	283.4 ± 12.82	163.6 ± 12.69	83.00 ± 9.850
HIF-1α	0.1533 ±0.03079	0.9729 ±0.06239	0.7501 ± 0.006199	0.1446 ± 0.07263
Crh	0.2495 ± 0.1264	0.9468±0.07509	0.7232 ± 0.06287	0.3093 ± 0.1387
Nr3c1	0.8801 ± 0.05706	0.1843 ± 0.06299	0.5870 ± 0.06238	0.8860 ± 0.06857
Nr3c2	0.9224 ± 0.07002	0.2195 ± 0.06199	0.6530 ± 0.02424	0.9537 ± 0.1268
ACTH	710.1 ± 57.79	316.0 ± 31.23	513.3 ± 39.01	702.5 ± 63.35
CORT	110.1 ± 10.29	41.80 ± 4.434	79.90 ± 6.377	107.9 ± 11.23
